# Circulating MicroRNAs associated with Bronchodilator Response in Childhood Asthma

**DOI:** 10.21203/rs.3.rs-3101724/v1

**Published:** 2023-06-29

**Authors:** Rinku Sharma, Anshul Tiwari, Alvin T Kho, Alberta L. Wang, Upasna Srivastava, Shraddha Piparia, Brinda Desai, Richard Wong, Juan C Celedón, Stephen P Peters, Lewis J Smith, Charles G Irvin, Mario Castro, Scott T Weiss, Kelan G Tantisira, Michael J McGeachie

**Affiliations:** Brigham and Women’s Hospital and Harvard Medical School; Vanderbilt University; Brigham and Women’s Hospital and Harvard Medical School; Brigham and Women’s Hospital and Harvard Medical School; Yale University school of medicine; University of California San Diego and Rady Children’s Hospital; University of California San Diego and Rady Children’s Hospital; University of California San Diego and Rady Children’s Hospital; University of Pittsburgh, UPMC Children’s Hospital of Pittsburgh; Wake Forest University; Northwestern University; University of Vermont; University of Kansas School of Medicine; Brigham and Women’s Hospital and Harvard Medical School; University of California San Diego and Rady Children’s Hospital; Brigham and Women’s Hospital and Harvard Medical School

**Keywords:** Bronchodilator response, microRNA, Asthma

## Abstract

**Rationale:**

Bronchodilator response (BDR) is a measure of improvement in airway smooth muscle tone, inhibition of liquid accumulation and mucus section into the lumen in response to short-acting beta-2 agonists that varies among asthmatic patients. MicroRNAs (miRNAs) are well-known post-translational regulators. Identifying miRNAs associated with BDR could lead to a better understanding of the underlying complex pathophysiology.

**Objective:**

The purpose of this study is to identify circulating miRNAs associated with bronchodilator response in asthma and decipher possible mechanism of bronchodilator response variation.

**Methods:**

We used available small RNA sequencing on blood serum from 1,134 asthmatic children aged 6 to 14 years who participated in the Genetics of Asthma in Costa Rica Study (GACRS). We filtered the participants into high and low bronchodilator response (BDR) quartiles and used DeSeq2 to identify miRNAs with differential expression (DE) in high (N= 277) vs low (N= 278) BDR group. Replication was carried out in the Leukotriene modifier Or Corticosteroids or Corticosteroid-Salmeterol trial (LOCCS), an adult asthma cohort. The putative target genes of DE miRNAs were identified, and pathway enrichment analysis was performed.

**Results:**

We identified 10 down-regulated miRNAs having odds ratios (OR) between 0.37 and 0.76 for a doubling of miRNA counts and one up-regulated miRNA (OR=2.26) between high and low BDR group. These were assessed for replication in the LOCCS cohort, where two miRNAs (miR-200b-3p and miR-1246) were associated. Further, functional annotation of 11 DE miRNAs were performed as well as of two replicated miRs. Target genes of these miRs were enriched in regulation of cholesterol biosynthesis by SREBPs, ESR-mediated signaling, G1/S transition, RHO GTPase cycle, and signaling by TGFB family pathways.

**Conclusion:**

MiRNAs miR-1246 and miR-200b-3p are associated with both childhood and adult asthma BDR. Our findings add to the growing body of evidence that miRNAs play a significant role in the difference of asthma treatment response among patients as it points to genomic regulatory machinery underlying difference in bronchodilator response among patients.

**Trial registration:**

LOCCS cohort [ClinicalTrials.gov number: NCT00156819], GACRS cohort [ClinicalTrials.gov number: NCT00021840]

## Background

Asthma is a heterogeneous disease that affects over 300 million people worldwide (1). It is characterized by chronic airway inflammation and a clinical history of wheezing, coughing, chest tightness, and shortness of breath that varies with time and intensity, as well as expiratory airflow limitation that is variably reversible with inhaled bronchodilators (2).

Albuterol, a short-acting beta-2 agonist (SABA) and bronchodilator, is one of the most used asthma medications in both children and adults (3). β2-agonists promote bronchodilation by activating β2-adrenergic receptors (β2ARs) on airway smooth muscle cells, resulting in decreased bronchoconstriction via increases in cyclic adenosine monophosphate (cAMP) and protein kinase A (PKA) (4). This is a physiological response that involves interaction between several cells and tissues such as inflammatory (5) and various airway cell types: the epithelium (6); smooth muscle cells (7); and cells of the autonomic nervous system (8).

Bronchodilator response (BDR) assesses the difference in FEV1 before and after the administration of a SABA. SABAs have variable efficacy among patients and BDR testing can be used to assess such effectiveness (9). Thus, studying BDR may provide insight into both the pathophysiology and pharmacogenetics of asthma.

High variability in BDR among individuals and populations has been described and is in part due to environmental and genetic factors (10–12). Estimates of BDR heritability range between 31–92% (13–15), and genome-wide association studies (GWASs) and whole-genome sequencing studies have identified susceptibility genes for BDR in subjects with asthma. Such genes include those for the β2-adrenergic receptor (*ADRB2*) (16), adenylyl cyclase type 9 (*ADCY9*) (17), corticotrophin-releasing hormone receptor 2 (*CRHR2*) (18), arginase 1 (ARG1) (19), and Spermatogenesis Associated Serine Rich 2 Like (*SPATS2L*) (20). These also highlighted several biological pathways that are likely to be involved in BDR control (e.g., Erk1/2 signal transduction, PI3K/Akt signal transduction, and nitric-oxide (NO) signaling pathway) (21, 22). Previously, pharmaco-metabolomics of bronchodilator response in asthma were also studied (23, 24).

MicroRNAs (miRNAs) are small non-coding RNA molecules that have sizes ranging from 18 to 22 nucleotides which act as post-transcriptional regulators of target gene expression (25) and have emerged as key regulators of epithelial cell and inflammatory processes (26). Circulating miRNAs have been suggested as biomarkers for a number of conditions, and have been shown to be important in a number of inflammatory-mediated processes (27, 28), including asthma (29). We hypothesized that miRNAs regulate bronchodilator response, a key intermediate phenotype of asthma. To test this hypothesis, we examined the relation between serum miRNAs and bronchodilator response in children with asthma and then attempted to replicate the significant findings in a cohort of adults with asthma.

## Methods

### Study Population

Subject recruitment and study procedures for the Genetics of Asthma in Costa Rica Study (GACRS) have been described in detail elsewhere (30, 31). In brief, the GACRS included 1165 Costa Rican children with asthma aged 6 to 14 years who were recruited between February 2001 and July 2011. Asthma was defined as physician-diagnosed asthma and having either at least two respiratory symptoms (wheezing, coughing, or dyspnea) or a history of asthma exacerbations in the previous year. Further, all participants had a high probability of having at least six great-grandparents born in Costa Rica’s Central Valley, as determined by a genealogist based on each of the child’s parents’ paternal and maternal last names. Participants in the study completed a protocol that included a questionnaire on respiratory and general health that was slightly modified from one used in the Collaborative Study on the Genetics of Asthma (32). Spirometry was performed using a Survey Tach Spirometer (Warren E. Collins, Braintree, MA, USA) in accordance with American Thoracic Society guidelines. Airway hyperresponsiveness was measured as a provocative dose of methacholine resulting in 20% reduction of FEV_1_. The study was approved by the Institutional Review Boards (IRBs) of the Hospital Nacional de Niños (San José, Costa Rica) and Brigham and Women’s Hospital (BWH; Boston, MA, USA). The current analysis was approved by BWH’s IRB (# 2017P001799).

### Replication Population

The Leukotriene modifier Or Corticosteroids or Corticosteroid-Salmeterol trial (LOCCS) (ClinicaTrials.gov— NCT00156819) has been previously described in detail (33). In summary, LOCCS enrolled 500 individuals with mild asthma to find the best step-down therapy for those who were well-controlled on low-dose ICS. The trial was held between 2003 and 2005. Fluticasone 100 g twice daily or fluticasone/salmeterol 100 g/50 g once daily provided better asthma control than montelukast alone, as measured by fewer treatment failures, fewer nocturnal awakenings, improved lung function, and higher asthma control questionnaire (ACQ) scores. The treatment was given in a double-blind fashion for 16 weeks. The time to treatment failure was the primary outcome. FEV1 and Bronchodilator assessment were measured at enrollment during the run-in period of ICS treatment. Participants in the LOCCS were mostly white, but there were a few participants from other racial or ethnic groups (e.g., Black, and Asian [see Table 1]).

### Primary Outcome

Bronchodilator response (BDR) testing was performed according to American Thoracic Society criteria (34). BDR was calculated as the percent change in FEV1 in response to administration of 200 μg of inhaled albuterol, as (([post-BD FEV1 – pre-BD FEV1]/pre-BD FEV1) x 100). Percent-predicted FEV1 (ppFEV1) was computed using expected FEV1 formulae for age, sex, height, and race according to Hankinson et al. (35).

### Sample Sequencing and Quality Control

We performed small RNA sequencing on serum from 1,134 GACRS samples of asthma patients and 390 samples of asthma patients in LOCCS. Both cohorts were sequenced following the same protocols (36). In brief, small RNA-seq libraries were prepared by using the Norgen Biotek Small RNA Library Prep Kit (Norgen Biotek, Therold, Canada) and sequenced on the Illumina NextSeq 500 platform. The ExceRpt pipeline was used for quality control (QC) of the RNA-seq data (37). The samples with less than 100k mapped reads were removed. miRNAs with less than five mapped reads in at least 50% of subjects were removed. We used the guided Principal Component Analysis (gPCA) (38) package for the identification of batch effects in GACRS and LOCCS.

### Identification of Differentially Expressed miRNAs and Statistical Approach

To decrease the intrinsic difference of BDR, high versus lowest quartiles of BDR were considered (named as high and low BDR group) and identified differentially expressed miRNAs between the high versus low BDR group using DESeq2 (39), which uses negative binomial regression, with a Benjamini–Hochberg false discovery rate (FDR) correction for multiple testing. A significance threshold of 10% FDR was used. The analysis was performed with adjustment for age, sex, use of inhaled corticosteroids (ICS) in the previous year and, baseline (pre-bronchodilator) percent predicted FEV1. Negative binomial regression was used to obtain estimates of effect size (betas and Odds Ratios) for a doubling of miRNA counts.

Top DE miRNAs were assessed for association with LOCCS high vs low BDR using DESeq2 and adjusted for age, sex, race, and baseline percent predicted FEV1.

Clinical and demographic features were compared using a Chi-square test for dichotomous variables and a t-test for continuous variables.

### Functional Annotation of Differentially Expressed miRNAs

Putative target mRNA transcripts were identified for 11 DE miRNAs between high and low BDR group and two replicated miRNAs separately using the miRecords version 4 (40), TarBase version 8 (41), and miRTarBase version 7.0 (42) databases using multiMiR package version 1.16 (43) with only the experimentally validated target mRNA transcripts considered. The union of targets of 11 DE miRNAs as well as targets of each DE miRNA separately were used for Reactome database pathway (44) analyses through the clusterProfiler package version 4.2.2 (45). We considered a Bonferroni adjusted p-value threshold of < 0.05 and a gene count of 3 or more to indicate significant enrichment of targeted genes for each biological pathway.

## Results

### Cohort Characteristics

Of 1,165 children with asthma from the GACRS, serum samples were available for 1,134 children. Of these, 555 GACRS participants fell in the high (N = 277) and low (N = 278) BDR group with − 3.82% and 17.6% average BDR values respectively (Table 1A). In terms of age and gender distribution, both groups (high vs low BDR) were similar with no significant difference (p-value ≥ 0.70 in both instances). Compared with children with the low BDR, those with the high BDR were significantly more likely to have used ICS in the previous year and to have lower percent predicted pre-BD FEV1 and higher eosinophil count and airway responsiveness.

Similar trends were seen in the LOCCS replication cohort (Table 1B): both the groups were similar in terms of gender and race distribution but there was a significant (p-value = 0.01) trend in age distribution. The high BDR response group participants were younger than the low BDR response group. In this cohort also, the participants in high-response group had lower baseline ppFEV1 as compared to low-response group (Table 1B) though this was not significant for blood eosinophil count or airway hyperresponsiveness.

### Sample Sequencing and Quality Control

In the GACRS, after filtering out samples with less than 100k mapped reads and miRNAs with less than five mapped reads in at least 50% of subjects, we had 1,134 participants with 317 miRNAs. In the LOCCS cohort, after filtering samples with less than 100k mapped reads, 24 samples dropped out. There were nine participants with missing bronchodilator response values. This left 357 participants with 179 miRNAs having more than five mapped reads in at least 50% of participants. Both the cohort miRNA expression data had no significant batch effect (p-value = 1).

### Identification of Differentially Expressed miRNAs

**Figure 1:**
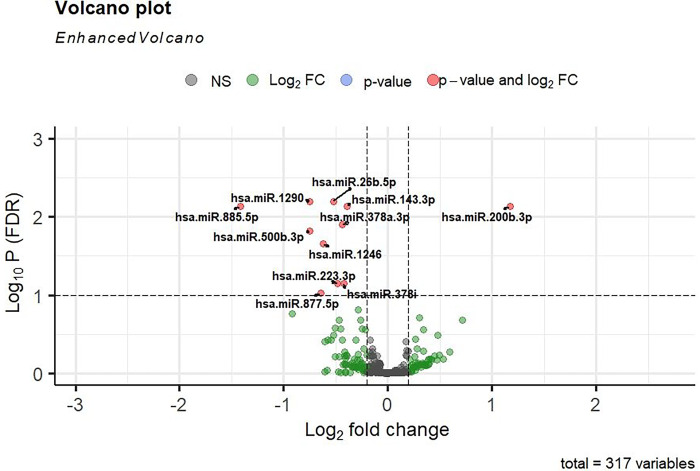
Volcano plot for differential expression of miRNA between high and low BDR in the GACRS. Y-axis: represents the multiple testing corrected (FDR) p-value.

**Figure 2:**
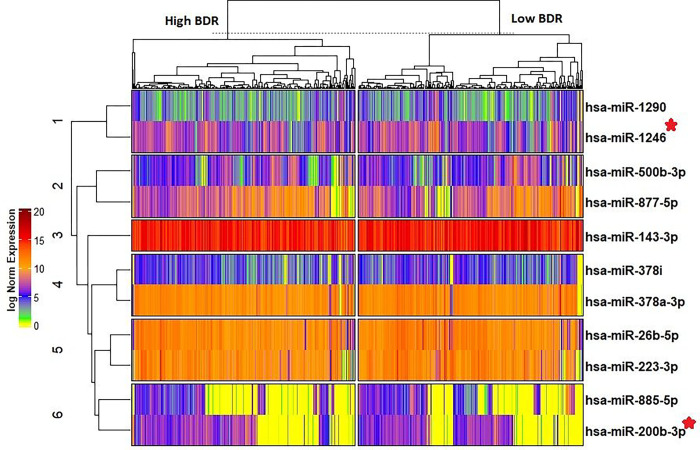
Clustered heatmap of all 11 differentially expressed miRs in the GACRS across conditions. DESeq2 normalized expression counts with shifted logarithm transformation was used. The heat map was created using unsupervised hierarchical clustering, and the distance metric was Pearson correlation. *Marked miRNAs were replicated in the LOCCS cohort.

The differentially expressed (DE) miRNA analysis was performed with high (N = 277) vs low (278) BDR-response groups in GACRS and identified 11 DE miRNAs with 10 down-regulated expression (odds ratios (OR) between 0.37 and 0.76) and one up-regulated expression (OR = 2.26) (Table 2A & Fig. 1) at 10% FDR. A clustered heatmap of 11 DE miRNAs is shown in Fig. 2. These 11 miRNAs were tested for differential expression in two BDR-response groups in another independent asthma cohort of adults and identified two miRNAs (miR-1246 and miR-200b-3p) with same direction of effect at p-value < 0.05 (Table 2B).

### Identification of Putative Targets and Functional Assessment of Differentially Expressed miRNAs

**Figure 3:**
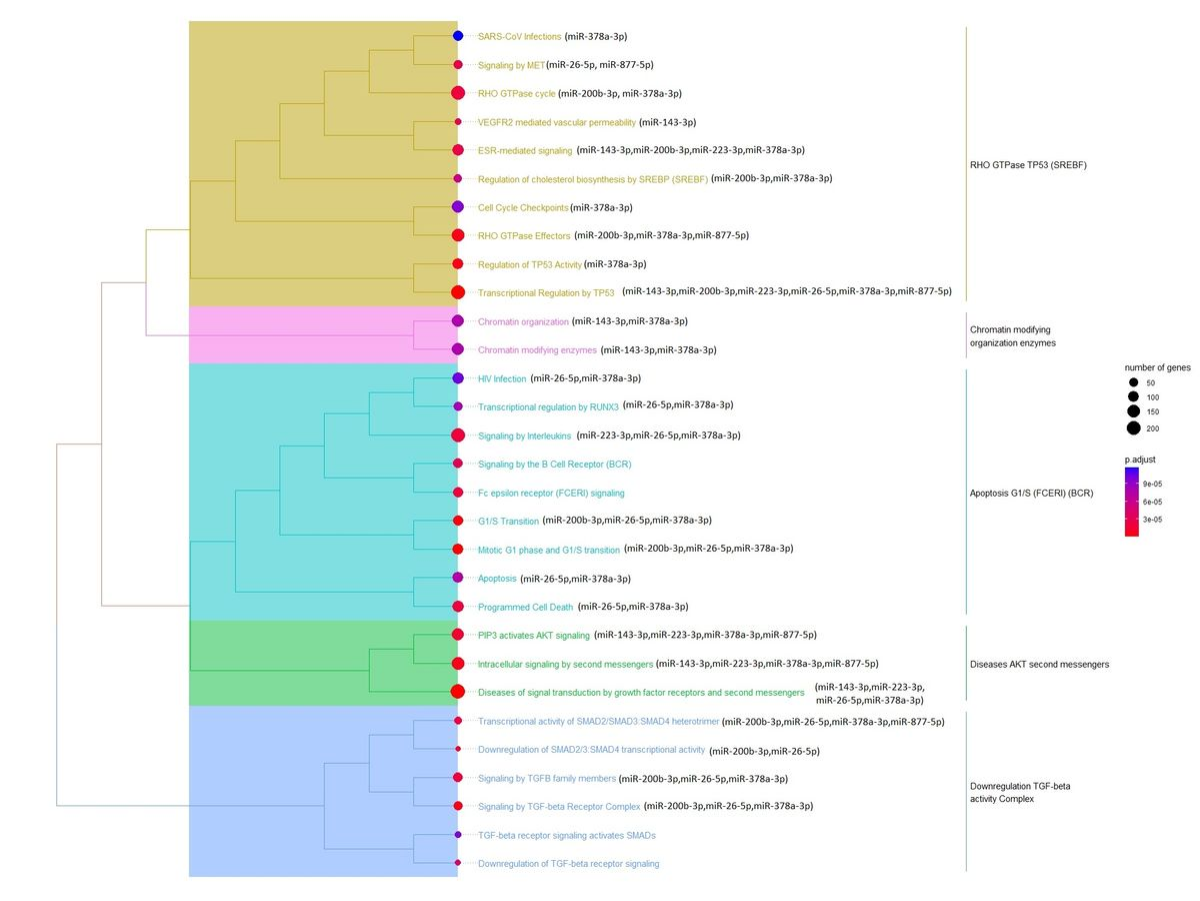
Reactome pathways enriched for 11 DE miRNAs in the GACRS at 5% FDR cut-off. The target genes were identified using Micro T-CDS, TarBase, and Target Scan databases. The pairwise similarities of the enriched terms calculated by the pairwise_termsim function using Jaccard’s similarity index (JC) and the agglomeration method ward.Din is used for clustering in R. If a pathway was found to be enriched with a specific DE miRNA’s target gene, the DE miRNA name is written next to it.

**Figure 4:**
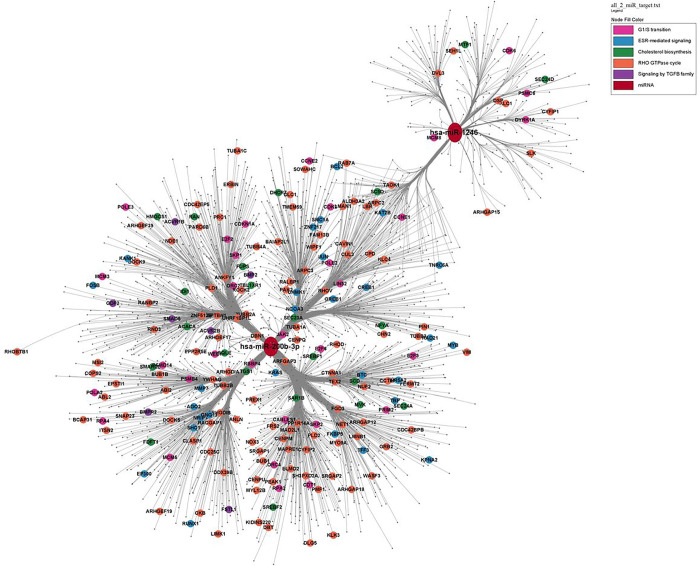
miRNA-target gene network between two replicated miRNAs. Nodes with different colors represent the genes in selected Reactome pathways.

We found 6,245 putative target mRNA transcripts for 11 DE miRNAs that were reported in databases using experimental approaches and performed functional pathway enrichment analysis of Reactome biological pathways (44) using the clusterProfiler R package (45). We also performed functional pathway enrichment analysis on each of the DE miRNA’s targets separately. The top 30 enriched pathways are shown in Fig. 3. Regulation of cholesterol biosynthesis by SREBPs, ESR-mediated signaling, G1/S transition, RHO GTPase cycle, and Signaling by TGFB family pathways were among the top enriched pathways. It was also observed that miR-200b-3p target genes were also enriched in these pathways (Fig. 4).

## Discussion

Asthma is a common disease of the airway, causing expiratory airflow limitation that is partially reversible with inhaled bronchodilators. Bronchodilators are the first-line treatment for asthma, and they work by acting on beta-2-adrenergic receptors on airway smooth muscle (ASM) cells in the lower respiratory tract, allowing muscle relaxation and bronchodilation (46) as well as inhibits liquid accumulation and mucus section into the lumen(47–49). In this study, we tried to identify serum miRNAs as indicator of BDR in a cohort of children with asthma (GACRS) followed by replication study in an adult asthma (LOCCS) cohort. We found differences in ICS use, baseline FEV1, and PD20 among participants with high vs. low BDR in the GACRS cohort. Sometimes, BDR is greater in patients with lower starting, since they have more to gain from a bronchodilator FEV1 (regression beta = −.41, p-value < 10^− 15^). We have attempted to correct for this effect by including baseline FEV1 as a covariate in our analysis, so that miRNAs associated with BDR should be more indicative of the airway’s plasticity rather than the magnitude of lung function deficit. ICS use increases pre-BDR FEV1 (50, 51) and would then decrease BDR (50), however, we noticed that ICS use was higher in patients with high BDR group. This may be due to confounding by indication, with patients with more serious disease and lower lung function requiring ICS therapy. Although PD20 differed by BDR response group, we did not include this as a covariate since it was anti-correlated with BDR, and inclusion in our model would result in decreased power.

We performed differential expression analysis using DeSeq2 to identify miRNAs associated with high vs low BDR and adjusting model for age, sex, use of inhaled corticosteroids (ICS) in the previous year and, baseline (pre-BDR) percent predicted FEV1. We found 11 miRNAs significantly associated with high vs low BDR in a study of Costa Rican children with asthma. In subjects with a high bronchodilator response, 10 of these 11 miRNAs were down-regulated, while one was up-regulated. Two of these miRNAs (miR-1246 and miR-200b-3p) were validated as being significant in the LOCCS cohort and regulated in the same direction, *i.e*., miR-1246 was down-regulated and miR-200b-3p was up-regulated in subjects with high BDR. These 11 miRNAs, and particularly miRs 200b-3p and 1246, may be indicative of general biological state that leads to difference in BDR.

The two replicated miRNAs were previously reported as potential biomarkers for respiratory diseases. miR-1246 has been reported to predict response to benralizumab in severe eosinophilic asthma (52), to distinguish healthy subjects from those with asthma (53), and to differentiate asthma from COPD or asthma–COPD overlap (ACO) along with two other miRNAs in a logistic regression model (54). Over-expression of miR-200–3p has been shown to reduce airway inflammation, mucus hypersecretion, and remodeling in asthma (55). Another study also suggested adenosine to inosine (A-to-I) edited sites in miR-200–3p in lower airway cells is associated with moderate-to-severe asthma (56). The putative target identification of these miRNAs revealed that miR-200b-3p regulates the expression of *SPATS2L*, a gene that was previously reported as a BDR gene (20) and miR-1246 regulates *ADCY9*, another BDR gene (17, 57). DIANA-miTED: a microRNA tissue expression database (58) also shows that these replicated miRNAs, namely miR-200b-3p (42986.7 RPM) and miR-1246 (147.7 RPM), are expressed in the bronchus.

Both gene targets of all 11 DE miRNAs in aggregate and gene targets of the two replicated miRNAs separately were enriched in regulation of cholesterol biosynthesis by SREBPs, ESR-mediated signaling, G1/S transition, RHO GTPase cycle, and signaling by TGFB family pathways (Figs. 3 & 4).

Regulation of cholesterol biosynthesis by the SREBPs pathway promotes cholesterol accumulation through uptake (low-density lipoprotein receptor) and synthesis (e.g., hydroxymethylglutaryl coenzyme A reductase) in macrophages and other cells (59). Recent findings indicate that cholesterol trafficking and inflammation are associated in the lung (60–63). In the present study we found that the target genes of DE miRNAs were enriched in this pathway, which may indicate the role of BDR-responsive miRNAs in cholesterol trafficking and inflammatory response in asthma. This is of interest as there are studies that link BDR to the presence of inflammation.

RHO GTPase pathway is known to regulate many essential cellular processes, including actin dynamics, gene transcription, cell-cycle progression and cell adhesion (64). We found that miR-200b-3p regulates the expression of ROCK2 gene that encodes Rho-kinase, known to play a role in regulating mucus overproduction (65), airway smooth muscle (ASM) tone (66) and ASM cytoskeletal stiffness (67). Further, ROCK2 expression is increased in ASM and pulmonary blood vessels in human asthma (68). This indicates a possible role of miR-200b-3p in regulating bronchoconstriction. Further exploration of the mechanisms by which miR-200b-3p and its target gene ROCK2 affect BDR may be worth pursuing.

**Figure 5:**
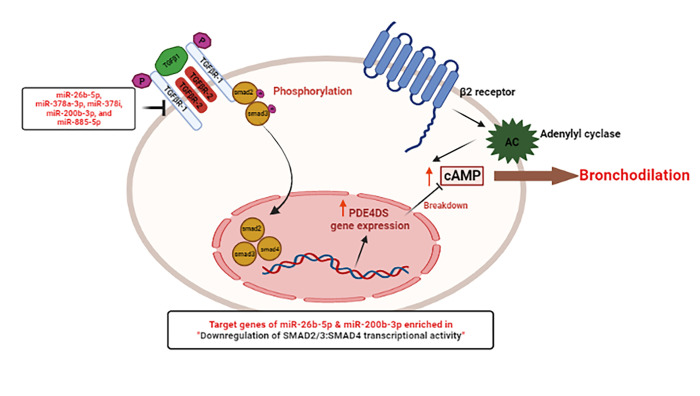
Bronchodilator response resistance mechanism: β2-Agonists cause relaxation by activating the β2-adrenoceptor, which then activates adenylyl cyclase (AC) to increase cAMP and cause bronchodilation. The increased resistance to β2-agonist-induced bronchodilation in asthmatics may be mediated by the effects of transforming growth factor (TGF)-β1. TGF-β1 activates the TGF-β receptor, causing phosphorylation of the transcription factors Smad2 and Smad3, which then translocate to the nucleus and form a complex with Smad4. This complex increases the expression of the PDE isomer PDE4DS, which leads to greater cAMP breakdown and, as a result, less bronchodilation. P = phosphorylation (*Wortley et al*., 2019). We found that BDR responsive DE miRs: miR-26b-5p, miR-378a-3p, miR-378i, miR-200b-3p, and miR-885-5p putatively target (TGFβ1, TGFβ2, TGFBR1, TGFBR2, and TGFBR3) genes encoding TGF-β and TGF-β receptor encoding, and miR-26-5p & miR-200b-3p putative targets were enriched in pathway associated with downregulation of SMAD2/3: SMAD4 transcriptional activity (FDR=1.09 × 10^−5^).

TGFB family pathway is known to play a role in epithelial shedding, mucus hyper-secretion, angiogenesis, airway hyperresponsiveness, ASMC hypertrophy and hyperplasia in an asthmatic mouse model (69–71). Previously, it has been reported that eosinophils constitute a major source of TGF-β in asthmatic airways (72, 73). In this study, participants with a high bronchodilator response had a higher eosinophil count than those with a low bronchodilator response (Table 1). Previous investigations suggest that TGF-β1 may play a role in the development of resistance to bronchodilators in asthma by reducing the efficacy of β2-agonists and by inducing PDE4D gene expression in a Smad2/3-dependent pathway manner (74–77). The DE miRNAs (miR-26b-5p, miR-378a-3p, miR-378i, miR-200b-3p, and miR-885-5p) were found to regulates the expression of *TGFB1*, *TGFB2*, *TGFBR1*, *TGFBR2*, and *TGFBR3* genes encoding TGF-β and TGF-β receptor (Supplementary Table S1). Additionally, the target genes of DE miRNAs were found to be enriched in TGFB pathway and pathway associated with downregulation of SMAD2/3: SMAD4 transcriptional activity (Fig. 3), showing possible role of DE miRNAs in regulation of TGF-β associated pathway and thus involved in smooth muscle remodeling (Fig. 5). TGF-β works upstream of the RHO GTPase pathway, TGF-β activates RhoA/rho-associated protein kinase (ROCK), and the cross-talk between these two pathways promotes airway remodeling (78) and mucus formation (69).

Strengths of our study include leveraging a large cohort of childhood asthmatics with circulating miRNA sequencing data and careful spirometric evaluation. That two of the identified miRNAs were able to replicate in an adult asthma population, despite etiological differences between childhood and adult asthma, gives weight to their importance in determining efficacy of SABAs as rescue inhalers. Weaknesses of our study include the retrospective study design and inability to assess miRNA differences in airway smooth muscle cells. We anticipate that future work into ASM miRNAs would provide additional biological insight into differences in BDR.

## Conclusion

In summary, we have identified differential expression of 11 miRNAs by bronchodilator response in children with asthma, that these miRNAs influence biological pathways associated with inflammatory response, airway smooth muscle cell contraction and airway remodeling, and that two of these miRNAs were replicated in another cohort of adults with asthma. Our findings add to the growing body of evidence that miRNAs play an important role in asthma treatment response differences among patients.

## Figures and Tables

**Table 1 A.** Baseline Epidemiologic and Clinical Characteristics of the GACRS cohort data

GACRS	Low BDR	High BDR	P-value
	(N = 278)	(N = 277)	
**Gender**			
Male	166 (59.7%)	160 (57.8%)	0.704
Female	112 (40.4%)	117 (42.2%)	
**Age (years)**			
Mean (SD)	9.02 (1.81)	8.99 (1.87)	0.858
Median [Min, Max]	8.72 [5.44, 14.2]	8.67 [6.02, 15.2]	
**Bronchodilator Response as % of baseline FEV1**			
Mean (SD)	−3.82 (3.74)	17.6 (11.5)	< 0.001
Median [Min, Max]	−2.81 [−24.6, 0.131]	14.0 [8.75, 89.7]	
**Log10 Eosinophil**			
Mean (SD)	2.54 (0.414)	2.70 (0.375)	< 0.001
Median [Min, Max]	2.63 [1.00, 3.35]	2.76 [1.00, 3.41]	
Missing	9 (3.2%)	6 (2.2%)	
**Vitamin D (ng/ml)**			
Mean (SD)	38.3 (12.4)	37.3 (13.1)	0.52
Median [Min, Max]	37.8 [16.6, 98.1]	35.7 [16.6, 97.7]	
Missing	137 (49.6%)	129 (46.6%)	
**Airway hyper-responsiveness**			
Mean (SD)	2.36 (2.69)	1.43 (2.17)	< 0.001
Median [Min, Max]	1.40 [0.104, 15.5]	0.949 [0.0440, 16.6]	
Missing	107 (38.6%)	83 (30.0%)	
**ICS use history**			
No	156(56.1%)	110(39.7%)	< 0.001
Yes	122(43.9%)	167(60.3%)	
**% predicted Pre-BD FEV1**			
Mean (SD)	106 (16.7)	88.5 (16.4)	< 0.001
Median [Min, Max]	106 [34.6, 180]	89.1 [31.8, 131]	

**Table 1 B.** Baseline Epidemiologic and Clinical Characteristics of the LOCCS cohort data

LOCCS	Low BDR	High BDR	P-value
	(N = 89)	(N = 89)	
**Gender**			
Male	35 (39.3%)	39 (43.8%)	0.648
Female	54 (60.7%)	50 (56.2%)	
**Age (years)**			
Mean (SD)	32.4 (15.0)	27.0 (14.7)	0.0155
Median [Min, Max]	32.0 [7.00, 64.0]	25.0 [6.00, 66.0]	
**Race**			
White	56 (62.9%)	54 (60.7%)	0.726
Black	26 (29.2%)	29 (32.6%)	
Asian	3 (3.4%)	1 (1.1%)	
Other	4 (4.5%)	5 (5.6%)	
**BMI**			
Mean (SD)	27.6 (8.86)	25.6 (7.20)	0.0974
Median [Min, Max]	25.2 [13.9, 57.7]	24.0 [13.4, 50.5]	
**Bronchodilator Response as % of baseline FEV1**			
Mean (SD)	−0.339 (3.36)	15.5 (5.69)	< 0.001
Median [Min, Max]	0.733 [−15.6, 2.84]	14.0 [10.5, 46.2]	
**% Predicted Pre-BD FEV1**			
Mean (SD)	95.1 (12.8)	88.6 (7.37)	< 0.001
Median [Min, Max]	94.0 [80.0, 144]	87.0 [63.0, 107]	
**Pre-BD FEV1/FVC**			
Mean (SD)	0.796 (0.0701)	0.741 (0.0633)	< 0.001
Median [Min, Max]	0.800 [0.562, 0.993]	0.743 [0.575, 0.924]	
**log10 Eosinophil**			
Mean (SD)	2.30 (0.404)	2.26 (0.577)	0.666
Median [Min, Max]	2.36 [0, 3.02]	2.33 [0, 3.46]	
Missing	4 (4.5%)	6 (6.7%)	

**Table 2 A.** Significant up and downregulated miRNAs between high and low BDR group in the GACRS. Base Mean: normalized mean counts in reference group. Log2FC: base-2-fold change from High to Low BDR. p-value: computed with DESeq2. Beta and Odds Ratio from negative binomial regression are for a doubling of miR counts.

	DeSeq2				Negative Binomial Regression					
miRNA	baseMean	log2FoldChange	pvalue	FDR q value	beta	CI (Upper)	CI (Lower)	OR	pvalue	FDR q value
miR-1246	212.193	−0.623	0.00056	0.0221	−0.4343	−0.1839	−0.6853	0.6477	5.15E-04	6.29E-04
miR-1290	20.946	−0.749	0.00003	0.00637	−0.5424	−0.3119	−0.7735	0.5814	8.97E-06	9.87E-05
miR-143–3p	37205.6	−0.392	0.00007	0.0073	−0.2719	−0.1347	−0.4091	0.7619	6.66E-05	2.44E-04
miR-200b-3p	88.291	1.171	0.0001	0.0073	0.8175	1.2154	0.4211	2.2648	1.44E-04	3.18E-04
miR-223–3p	2825.22	−0.482	0.00226	0.0716	−0.3336	−0.1105	−0.5567	0.7163	2.93E-03	3.22E-03
miR-26b-5p	3143.41	−0.525	0.00004	0.00637	−0.3648	−0.1863	−0.5431	0.6943	4.48E-05	2.44E-04
miR-378a-3p	2849.76	−0.438	0.00024	0.0126	−0.3065	−0.1396	−0.4732	0.736	3.33E-04	5.23E-04
miR-378i	26.698	−0.421	0.00212	0.0716	−0.3703	−0.1697	−0.5707	0.6906	1.92E-04	3.52E-04
miR-500b-3p	145.668	−0.753	0.00033	0.015	−0.5193	−0.2	−0.8373	0.5949	4.28E-04	5.88E-04
miR-877–5p	976.479	−0.646	0.00326	0.094	−0.4519	−0.1466	−0.7573	0.6364	3.42E-03	3.42E-03
miR-885–5p	50.444	−1.419	0.00012	0.0073	−0.9849	−0.5166	−1.4523	0.3735	1.38E-04	3.18E-04

**Table 2 B.** List of replicated up and downregulated miRNAs in LOCCS cohort.

miRNA	baseMean	log2FoldChange	pvalue	FDR q value
miR-200b-3p	268.676	0.646	1.29E-02	1.53E-01
miR-1246	207.499	−1.312	8.17E-05	6.29E-03

## Data Availability

Sequencing data is available in Gene Expression Omnibus (accession pending).
